# A meta-analysis of the criterion-related validity of Session-RPE scales in adolescent athletes

**DOI:** 10.1186/s13102-023-00712-5

**Published:** 2023-08-12

**Authors:** Haochong Liu, Wenpu Yang, Haoyang Liu, Dapeng Bao, Yixiong Cui, Indy Man Kit Ho, Qian Li

**Affiliations:** 1 Sports Coaching College, Beijing Sport University, Beijing, China; 2 Sports Engineering Lab, School of Sports Engineering, Beijing Sport University, Beijing, China; 3 China Institute of Sport and Health Science, Beijing Sport University, Beijing, China; 4 Hong Kong Metropolitan University, Hong Kong, China; 5 Asian Academy for Sports and Fitness Professionals, Hong Kong, China

**Keywords:** Session-RPE, Adolescent, Criterion validity, Training load, Monitoring training

## Abstract

**Background:**

The objective of this study was to establish the criterion-related validity of the session-rating of perceived exertion (s-RPE) method in adolescent athletes.

**Methods:**

According to the Preferred Reporting Items for Systematic Reviews and Meta-Analyses (PRISMA 2020) guidelines, a meta-analysis (PROSPERO ID: CRD42022373126) was performed using Stata 15.1 software. Eight databases using the following terms: (‘s-RPE’ OR ‘Rating Perceived Exertion session’ OR ‘RPE session’ OR ‘RPE’ OR ‘Rate of Perceived Exertion’ OR ‘Rated of Perceived Exertion’) AND (‘Adolescen*’ OR ‘Youth*’ OR ‘Teen*’) AND (‘validity’ OR ‘correlation’ OR ‘concurrent validity’) were searched up to 2022. Articles meeting the inclusion criteria were screened and adopted the “Methodological Index for Non-Randomized Studies (MINORS)” to evaluate the risk of bias.

**Results:**

An initial 1798 studies using the s-RPE method were identified and finally, a total of 16 studies were included for further analysis. The relationship between assessment instruments CR-10 or CR-100 modified methods of s-RPE and the heart rate measures of these selected studies were calculated using correlation coefficient (*r* values) and Fisher’s z-score. A strong to very strong correlation between s-RPE and HR was observed (overall: *r* = 0.74; CR-10: *r* = 0.69; CR-100: *r* = 0.80). CR-100 scale (Fisher’s z = 1.09) was shown to have a higher criterion validity than that of the CR-10 scale (Fisher’s z = 0.85).

**Conclusion:**

Preliminary findings showed that s-RPE using either CR-10 or CR-100 scales can be used "stand-alone" for monitoring internal training load for children and adolescent athletes. Future studies should focus on whether CR-100 could better perform than CR-10 for junior and children athletes in different age groups and sports as well as the causes leading to potential scoring biases.

**Supplementary Information:**

The online version contains supplementary material available at 10.1186/s13102-023-00712-5.

## Background

Increasing numbers of adolescents are participating in sports training and competition, and it is believed that the increased availability of training and match-play can potentially increase the probability of success [1]. Meanwhile, based on the overloading concept, progressively increasing the training load (TL) is one of the keys to improving athletic performance. However, adding training intensity, time, or frequency without a structured and systematic manner will potentially increase the risk of injury and overtraining [2]. As adolescents are not simply “mini-adults”, it is important for youth athletes to undertake training in a suitable load that is compatible with their growth and maturation phase [3]. On the other hand, a poorly planned training program can lead to overtraining and in return induce physical and mental damage, or even lead to dropping out of sports. Due to the close association between excessively high training volumes and the occurrence of injuries among youth athletes, training and competition load monitoring are particularly pertinent [4].

There has been increasing evidence that proper TL management is important to progressively increase training volume among young athletes and promote long-term success in sport [5]. In addition to optimizing physical performance [6], reducing the incidence of injuries and illness [7, 8], and minimizing the risk of nonfunctional overreaching [9], TL monitoring also helps facilitate athletes and coaches in achieving their training goals and minimizing undesirable training outcomes [10]. Therefore, implementing TL monitoring during youth training plays an important role in their long-term athletic development.

The key to TL monitoring relies on the structured and proper arrangement of training intensity and volume, whereas an accurate and valid TL quantification method is vital in reflecting and manipulating those training parameters. Currently, TL is assessed primarily through internal measures (e.g., heart rate, HR) and external measures (e.g., Global Positioning Systems). However, since the external load is mainly measured by physical load (e.g., distance, speed, weight, etc.) [11], it tends to express an absolute training load rather than truly reflect the perceived load upon a given training program. Nevertheless, giving exercises or training programs with identical external loads to young athletes can induce varied physiological adaptations due to different maturation phases, individual differences, and perceived loads of children and adolescents. In this regard, young athletes undergoing rapid physiological changes are more likely to benefit from using internal TL monitoring.

Training impulse (TRIMP) and rating of perceived exertion (RPE) [12] are currently the most common methods to evaluate internal loads. TRIMP was first produced by Banister et al. [13] in 1975 and the Banister’s TRIMP [13] was calculated by training time and training intensity based on heart rate reserve. To enhance the easiness of TRIMP computation and take into account the intermittent exercise, Edwards et al. [14] introduced the heart rate zone in performing the calculation. In this regard, a weight factor of each heart rate zone is given whereas the TRIMP per each zone is acquired by multiplying the exercise time. The total TRIMP is the sum of all zone interval TRIMPs. As TRIMP measurement relies on HR monitoring devices, several limitations were identified such as high cost, the requirement of high technical proficiency, and the possibility of data loss due to technical error [11]. Furthermore, an obstacle in using HR methods to quantify internal training load (TL) in team sports like soccer is that HR transmitter belts are not allowed during official competitive matches. This limitation is significant because the internal training load resulting from a match may constitute a relatively high proportion of the overall weekly training load [15]. Conversely, the RPE method is more convenient and less expensive. However, since it is used during or immediately after training, it is more likely to overestimate fatigue due to the "fatigue is ongoing" effect. Foster subsequently proposed the session-RPE (s-RPE) method, based on a modified Borg category ratio CR-10 [16] and CR-100 [17], which was inspired by RPE and Banister’s TRIMP [18]. The calculation method is as follows:

1 $$\mathrm s-\mathrm{RPE}\;=\;\mathrm{training}\;\mathrm{duration}\;\times\;\mathrm{RPE}\;\mathrm{values}.$$

The s-RPE method is a more straightforward, non-invasive, and inexpensive method for TL monitoring meanwhile it can monitor both the external and internal load (e.g. mental fatigue perception) of training. Weekly TL, training monotony [19] (the variation in TL during the week), and training strain [10] (the overall stress during a week of training) can be calculated from TL data using s-RPE. In addition to providing athletes and coaches with quick and easy feedback on internal load conditions, the TL monitoring method using s-RPE allows timely adjustments of TL and training plans to enhance athletic performance and decreases the risk of sports injuries. Currently, this approach has been used in a wide variety of sports and strength and conditioning programs [10, 11, 20]. To acquire accurate data, Foster suggested that RPE values should be collected 30 min after a training session. More recent studies also suggested collecting s-RPE scores approximately 15 min after the end of the training session [21–24].

As these scales were developed and validated for adults, there may be some limitations when applied to children and adolescents [25]. For example, it has been reported that children’s understanding of the scale can affect their RPE scores [26]. Although numerous previous studies have adopted s-RPE in quantifying the TL of children and adolescents, to what extent the s-RPE-based TL can match the internal load (e.g. heart rate) is questionable. The effectiveness of RPE in children and adolescents is uncertain due to the limited research available and inconsistencies observed across studies [27]. Therefore, the purpose of this meta-analysis study was to characterize the strength of the relationship between internal training load indicators (heart rate and s-RPE) in adolescent athletes. This study aims to verify the validity of different s-RPR scales- (CR-10 and CR-100) by quantifying the correlation between the s-RPE and HR methods. This in return can inform coaches and researchers of the implications of using s-RPE to monitor TL in youth sports.

## Materials and methods

### Protocol and registration

A meta-analysis was conducted following the latest guidelines of the Preferred Reporting Items for Systematic Reviews and Meta-Analyses (PRISMA 2020) [28] and was registered with the PROSPERO database in General interest, on 15 November 2022 (CRD42022373126). Additional file 1 (Table S1) provides a description of the relevant adaptations.

### Eligibility criteria

We used PICOS (participants, intervention, comparators, outcomes, and study design) approach [29] for reference to rate studies for eligibility. Since the studies we chose were single-arm trials, only PIO can be used. Table 1 indicates our inclusion/ exclusion criteria.

**Table 1:** Modified selection criteria (PICOS) used in the meta-analysis

Category	Inclusion criteria	Exclusion criteria
POPULATION	Healthy athletes aged under 18 years old	Participants with concomitant pathology
INTERVENTION	Used both s-RPE scales (CR-10 or CR-100) and physical methods (TRIMP) to monitor the TL	TL monitoring without s-RPE and HR; without Edward’s TRIMP
COMPARATORS	Not used	Not used
OUTCOME	Provided data established a correlation between s-RPE and physiological outcome measures (TRIMP) as reference criteria	Lack of correlation
STUDY DESIGN	Single-arm trials	Other types of study design
OTHERS	Original and full-text studies written in English	Non-English

After removing duplicates, titles and abstracts from relevant articles were reviewed according to the inclusion and exclusion criteria. Full texts of all potentially relevant studies were obtained and processed for inclusion by two independent investigators (HC. L. and WP. Y). Data was blindly collected and recorded in a special worksheet. Study characteristics were recorded.

### Search strategy

We conducted a systematic literature search in the following databases PubMed, Web of Science, Ovid, Embase, Cochrane, EBSCO, Scopus, and ProQuest. Medical Subject Headings (MeSH) (adolescents) and free terms (s-RPE, RPE session, Rating Perceived Exertion session, RPE, Rate of Perceived Exertion, Rated of Perceived Exertion, young, teen*, validity, concurrent validity, correlation) were used. The Boolean operators AND and OR were also used. (The specific search strategy of different databases was in the Supplementary file 3). The search covered the period from the first publication to November 2022. In addition, reference lists of primary articles were reviewed.

### Data extraction and methods of the review

The following data were extracted from the included articles: authors with the year of publication, study sample size (sex (males and females)), age range (mean and standard deviation (SD)), exercise protocol (type, intensity, total time), exercise modality (the sports that athletes engage in), RPE familiarity (whether pre-familiar with RPE scales), s-RPE scale (e.g., CR-10 and CR-100), physiological criterion, validity criterion and results (i.e., the coefficient between s-RPE and validity criterion). Two reviewers independently appraised papers (WP. Y. and HC. L); a third reviewer (Q. L.) was consulted to resolve disputes. Contact was made with the principal authors if the data to be extracted could not be found. When no response was obtained from the principal authors, data were extracted from the figures of the studies using the WebPlotDigitizer software. Studies that were not written in English, adopted findings from adults, diseases, or animals, and did not use heart rate and s-RPE were excluded.

The PRISMA flowchart was adopted for illustrating the search results. In addition, we also manually checked the reference lists of eligible papers for the possibility of potentially suitable studies.

### Methodological quality and risk of bias assessment

Methodological quality (MQ) was assessed independently by two reviewers (HC. L and WP. Y) using the methodological index for non-randomized studies (MINORS) [30]. The MINORS is suitable for single-arm tests and is used to access the methodological quality of criterion-related validity studies. The total score for each study was used to rank the risk of bias as low (13–16), moderate (9–12), or high (0–8).

### Certainty assessment

Two evaluators (WP. Y. and HC. L) independently assessed the certainty of evidence using the Grading of Recommendations Assessment, Development, and Evaluation (GRADE) approach [31] through the GRADE PRO website (https://gradepro.org). GRADE specifies four categories: high, moderate, low, and very low, applied to a body of evidence.

### Statistical analysis

The relationships between s-RPE and TRIMP were computed using the Pearson correlation coefficient (*r*-value). The strength of relationships was classified as *r* ≤ 0.19, very weak; 0.2 ≤ *r* ≤ 0.39, weak; ≤ 0.4 *r* ≤ 0.59, moderate; 0.6 ≤ *r* ≤ 0.79, strong; *r* ≥ 0.8, very strong [32]. For correlation comparison, Fisher's r-to-z transformation was calculated for correcting the *r* values such that the normal distribution is satisfied. Therefore, after standardization z-values could be used for addition, subtraction, or comparison between data of different units or magnitude. The related formula is: z’ = 0.5[ln(1 + r)—ln(1-r)] [33]. The Cochran test was used to determine statistical heterogeneity among publications, and inconsistency was assessed using *I*^*2*^ statistics, defined as *I*^*2*^ = 100% (Q-DF)/Q, where Q is Cochran’s heterogeneity index and DF accounts for degrees of freedom. A value of 0% indicates a lack of heterogeneity, whereas a larger value shows the existence of heterogeneity. For analyses where *I*^*2*^ was below 50%, a fixed effects model was applied and, if *I*^*2*^ was above 50%, a random effects model was employed [34]. General criterion-related validity was established using calculated Fisher’s z between s-RPE and HR, and different scales of s-RPE were analyzed in the subgroup. A funnel plot [35] was constructed to evaluate publication bias, and the Begg and Egger’s tests were used. A 95% confidence interval (95% CI) was reported and the significance level was set at *p* < 0.05 according to the analysis conducted in Stata software version 15.1 (Stata Corp, College Station, TX, USA).

## Results

### Study selection

**Fig. 1:**
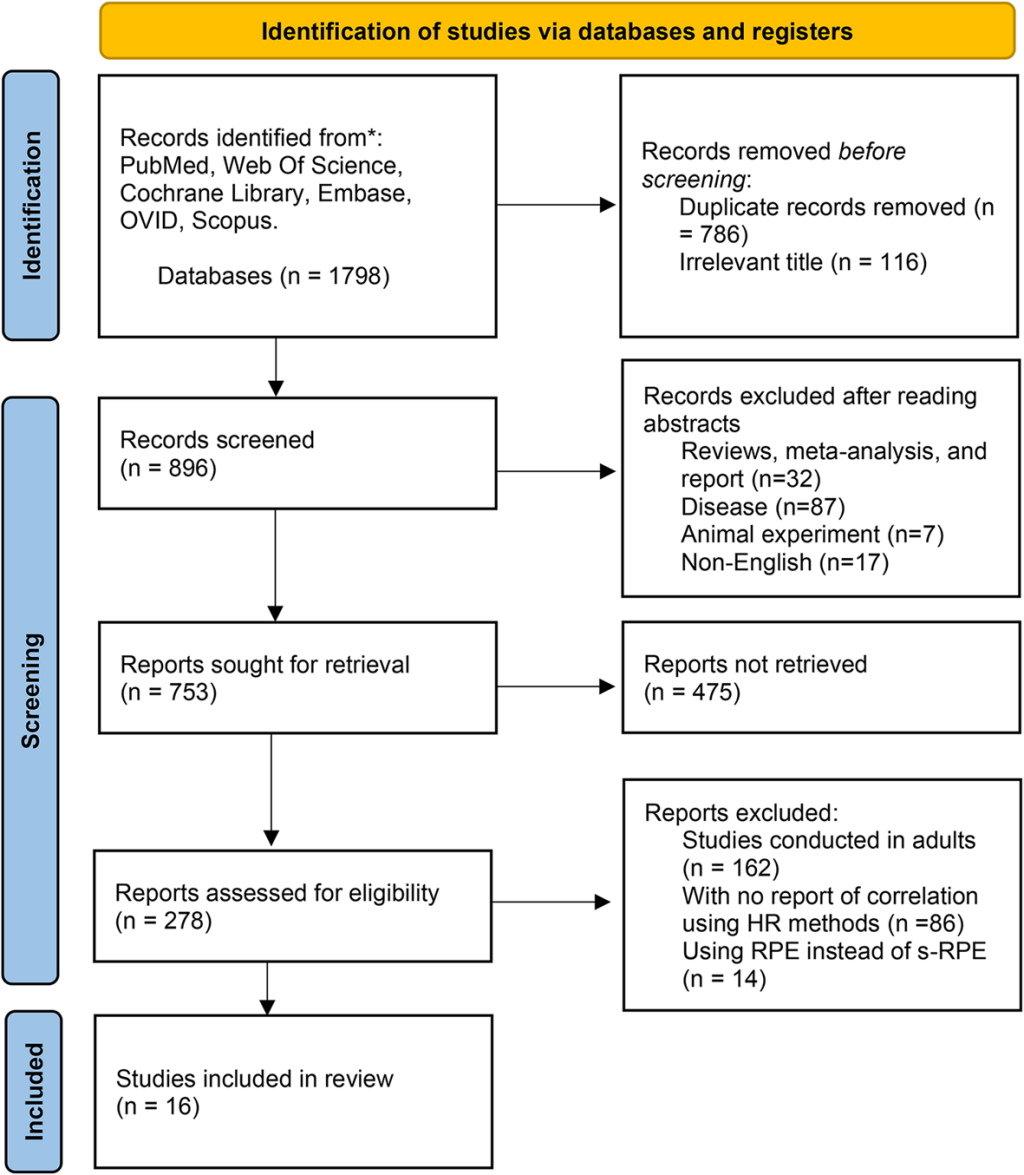
PRISMA flow diagram of Literature retrieval

The selection process is outlined in Fig. 1. A preliminary search identified 1798 potentially relevant studies. As a result of applying and screening for the inclusion and exclusion criteria described above, 16 studies met the inclusion criteria and were included in the meta-analysis.

### Study characteristics and quality assessment

Table 2 summarizes the key features of the papers that contributed to the systematic review findings concerning the authors, sample characteristics (i.e., sample size, age, sex, exercise type, and protocol), and end measure features (i.e., physiological index and RPE scale).

**Table 2:** Overview of selected reviews in the year and alphabetical order (*n* = 16)

**Author**	**Sample** **Size(M/F)**	**Age**	**Exercise** **Protocol**	**Exercise** **Modality**	**RPE** **Familiarity**	**s-RPE Scale**	**Physiological** **Criterion**	**Validity** **Criterion**	**Pearson’s *r* value**
Haddad et al. (2011) [36]	10 (10/0) youth competitive athletes	13.1 ± 2.4	308 training sessions	Taekwondo	2-week familiarization	CR-10	HR	Banister’s TRIMP Edwards’ TRIMP	*r* = 0.67 *r* = 0.63
Lupo et al. (2014) [37]	13 (13/0) elite-level athletes	15.6 ± 0.5	5–8(80 individual) training sessions (1:47:18 ± 0:15:26 h: min: s)	Water Polo	1-week familiarization	CR-10 (30 min after)	HR	Edwards’ TRIMP	*r* = 0.88 (*p* < 0.001)
Padulo et al. (2014) [38]	11 (11/0) Youth athletes	12.50 ± 1.84	110 sessions	karate	3-week familiarization	CR-10 (5 min after)	HR	Banister’s TRIMP Edwards’ TRIMP	*r* = 0.63 *r* = 0.79
Rodríguez-Marroyo et al. (2015) [25]	12 (12/0) Children players	11.4 ± 0.5	10 weeks 20 training sessions (83.2 ± 5.9 min)	soccer	1-month familiarization	CR-10 OMNI (30 min after)	HR	Edwards’ TRIMP	*r* = 0.17 (*p* = 0.335); *r* = 0.34 (*p* = 0.007)
Lupo et al. (2017) [22]	9 (4/5) pre-adolescent athletes	M12.0 ± 0.8F12.0 ± 0.7	17 (100 individual) training sessions	Taekwondo	2-week familiarization	CR-10 (30 min after)	HR	Edwards’ TRIMP	*r* = 0.71 (*p* < 0.001)
Lupo et al. (2017) [39]	6 (6/0) youth players	16.5 ± 0.5	15 (66 individual) training sessions (80 ± 26 min)	basketball	2-week familiarization	CR-10	HR	Edwards’ TRIMP	*r* = 0.85 (95%CI = 0.76–0.91 *p* < 0.001)
Scantlebury et al. (2017) [40]	29 (20/9) adolescent athletes	16.7 ± 0.8; 17.2 ± 0.4; 17.2 ± 0.8	14-week in-season training (397 sessions)	field-hockey, Rugby, soccer	NA	CR-10 (30 min after)	HR	Edwards’ TRIMP	*r* = 0.60 (95% CI = 0.47–0.710); *r* = 0.68 (95% CI = 0.59–0.7); *r* = 0.72 (95% CI = 0.62–0.8)
Mann et al. (2019) [41]	15 (15/0) adolescent distance runners	15.2 ± 1.6	69 exercise sessions	Treadmill run	familiarized	CR-10 (0 min, 15 min, 30 min) post-session	HR, blood lactate threshold	Edwards’ TRIMP	*r* = 0.74–0.89
Naidu et al. (2019) [42]	19 (19/0) State-level youth players	15 ± 1	3-month (460 training sessions)	football	4-week familiarization	CR100 (20 min after)	HR	Banister’s TRIMP Edwards’ TRIMP	*r* = 0.77 *r* = 0.84
Vahia et al. (2019) [43]	15 (15/0) professional youth players	16.7 ± 1	7 months (7 training sessions &1 match/ week)	football	1-month familiarization	CR-10 (30 min after)	HR	Edwards’ TRIMP	*r* = 0.64
Lovell et al. (2020) [44]	30 (30/0) elite players	16.7 ± 0.5	17 weeks 30 on-pitch training sessions competitive matches	soccer	2-week familiarization	CR100 (10–15 min post)	HR	Banister’s TRIMP Edwards’ TRIMP	*r* = 0.65–0.81
Lupo et al. (2020) [45]	15 (0/15) Elite players	16.7 ± 0.5	19 teams (268 individual) training sessions (102 ± 15 min)	basketball	≥ 1-week familiarization	CR-10 (30 min after)	HR	Edwards’ TRIMP	*r* = 0.59 (*p* < 0.00)
Cesanelli et al. (2021) [46]	8 (8/0) competitive junior category cyclists	16.2 ± 0.7	1 year Season (2–5 days/week)	cycling	NA	CR-10 (30 min after)	HR	Edwards’ TRIMP	*r* = 0.71 (*p* < 0.001)
Iannaccone et al. (2021) [47]	13 (13/0) youth players	15.9 ± 0.3	21 sessions	Beach handball	NA	CR-10 (30 min after)	HR	Edwards’ TRIMP	*r* = 0.74 *(p* = 0.78)
Serpiello et al. (2021) [48]	59 Elite academy players	12–17	1season (4 training sessions & 1 game /week)	football	≥ 1-season familiarization	CR-100	HR	Edwards’ TRIMP	U15: *r* = 0.78 (*p* = 0.08); U18: *r* = 0.83 (*p* = 0.05); U20: *r* = 0.84 (*p* = 0.08)
Maciel et al. (2022) [49]	14 (14/0) youth National Federation athletes	16.9 ± 1.1	12 training sessions	Handball	NA	CR-10 (30 min after)	HR	Edwards’ TRIMP	*r* = 0.36

*M* Male, *F* Female, *HR* Heart rate, *NA* Not available, *CR-10* Borg ratings of perceived exertion scale, *CR-100* Borg (0–100) RPE scales, *Edwards’ TRIMP and Banister’s TRIMP* External training monitoring indicators based on heart rate

Publication dates ranged from 2014 to 2022. The sample size of these studies ranged from 6 to 59 participants; 249 (89.6%) were male and 29 (10.4%) were female.

Exercise modality included football/soccer (*n* = 145, 52.2%), basketball (*n* = 21, 7.6%), field-hockey (*n* = 9, 3.2%), rugby (*n* = 10, 3.6%), handball (*n* = 27, 9.7%), water polo (*n* = 13, 4.7%), Taekwondo or karate (*n* = 30, 10.8%), and run or cycle (*n* = 23, 8.3%).

In relation to the reference criterion used, all studies recorded HR (*n* = 278, 100%). Additionally, 15 articles used the Borg scale from 0 to 10 (*n* = 170; 61.2%) and 3 articles used the Borg scale CR-100 (*n* = 108; 38.8%).

The bias scores of the studies included in this systematic review and meta-analysis ranged from 13 to 14 (low risk of bias) out of eight possible points (Table 3). All studies were classified as having a low risk of bias.

**Table 3:** Quality assessment

**MINORS**	**Box1**	**Box2**	**Box3**	**Box4**	**Box5**	**Box6**	**Box7**	**Box8**	**Score**
Haddad et al. [36]	2	2	2	2	2	2	2	0	14/16
Lupo et al. [37]	2	2	2	2	2	1	2	0	13/16
Padulo et al. [38]	2	2	2	2	2	2	2	0	14/16
Rodríguez-Marroyo et al. [25]	2	2	2	2	2	1	2	0	13/16
Lupo et al. [22]	2	2	2	2	2	1	2	0	13/16
Lupo et al. [39]	2	2	2	2	2	1	2	0	13/16
Scantlebury et al. [40]	2	2	2	2	2	2	2	0	14/16
Mann et al. [41]	2	2	2	2	2	1	2	0	13/16
Naidu et al. [42]	2	2	2	2	2	2	2	0	14/16
Vahia et al. [43]	2	2	2	2	2	2	2	0	14/16
Lovell et al. [44]	2	2	2	2	2	2	2	0	14/16
Lupo et al. [45]	2	2	2	2	2	2	2	0	14/16
Cesanelli et al. [46]	2	2	2	2	2	2	2	0	14/16
Iannaccone et al. [47]	2	2	2	2	2	1	2	0	13/16
Serpiello et al. [48]	2	2	2	2	2	2	2	0	14/16
Maciel et al. [49]	2	2	2	2	2	1	2	0	13/16

Box1: specific research purpose; Box2: the consistency of participants; Box3: Collection of the expected data; Box4: The endpoint index can accurately reflect the research purpose; Box5: Objectivity of the evaluation of the endpoint indicators; Box6: sufficient follow-up time; Box7: loss to follow-up% ≤ 5%; Box8: estimated sample size. 0: Not reported; 1: Reported but with inadequate information; 2: Reported and provided adequate information

### Results of meta-analysis

**Fig. 2:**
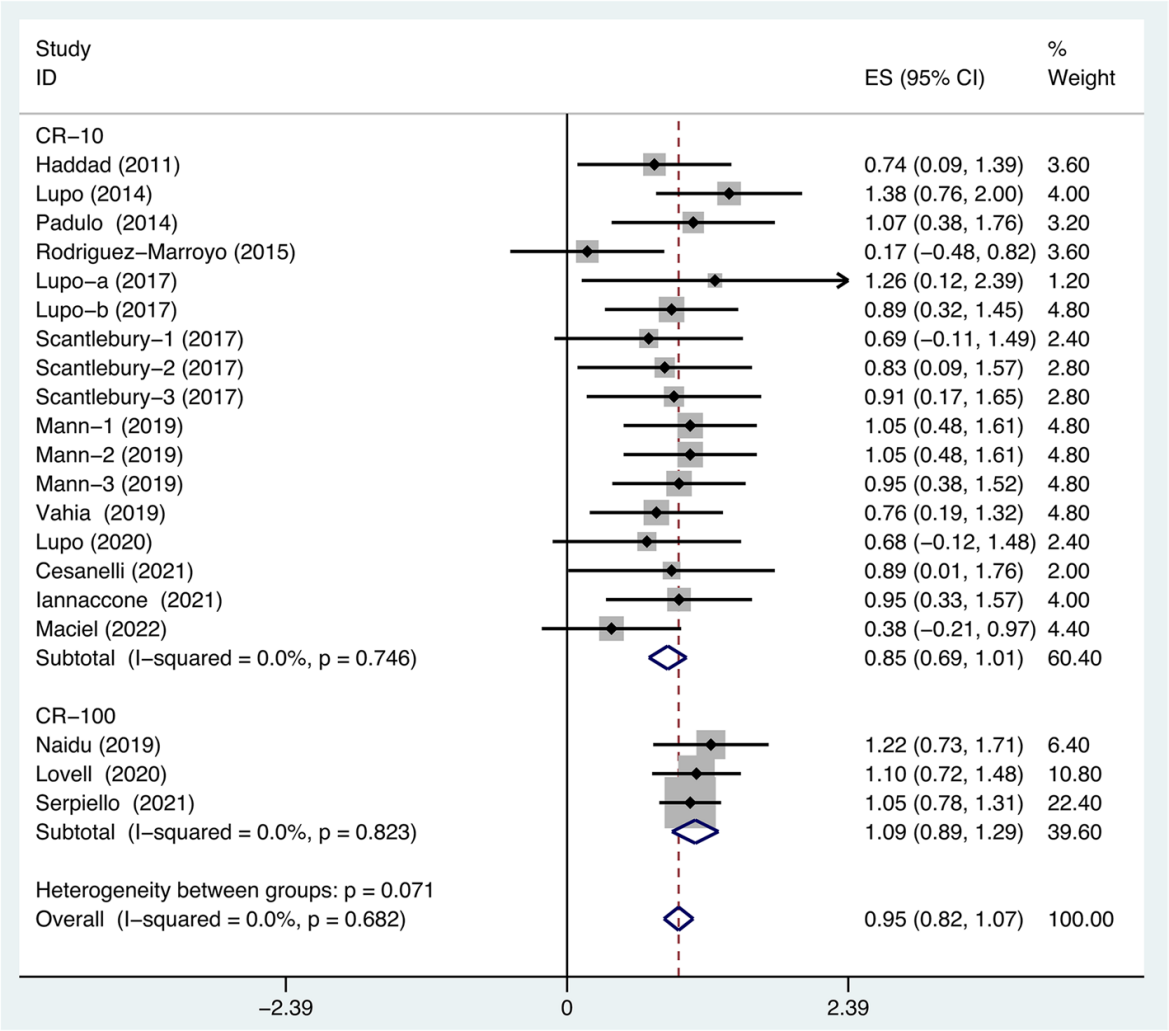
The subgroup of Meta-analysis of studies that used CR-10 and CR-100 considered heart rate as a reference criterion

A total of 15 studies evaluated the correlation between CR-10 and heart rate, involving 170 athletes, while a total of 3 studies evaluated the correlation between CR-100 and HR, involving 108 athletes. The heterogeneity test of CR-10 (*I*^*2*^ = 0%, *p* = 0.746) and CR-100 (*I*^*2*^ = 0%, *p* = 0.823) were performed and the fixed-effects model was used. Due to *I*^*2*^ = 0, we assume that the effect size is roughly the same for all populations, we omit the prediction interval. The results of the analysis showed that a weighted Fisher’s z between CR-10 and HR measures of 0.85 (95% CI: 0.69–1.01) and a weighted Fisher’s z between CR-100 and HR measures of 1.09 (95% CI: 0.89–1.29) (Fig. 2). The pooled Fisher’s z of s-RPE considering HR as the reference criterion is 0.95 (95% confidence interval: 0.82–1.07). We used Excel tools to transfer Fisher’s z to Pearson’s r, CR-10 vs HR (*r* = 0.69, 95% CI: 0.60–0.77), CR-100 vs HR (*r* = 0.80, 95% CI: 0.71–0.86), s-RPE vs HR (*r* = 0.74, 95% CI: 0.68–0.79). According to the GRADE approach, the certainty of the evidence was established as “high”, which suggests that the authors are highly confident in the association (see Supplementary file Figure S1).

### Sensitivity analysis and publication bias

**Fig. 3:**
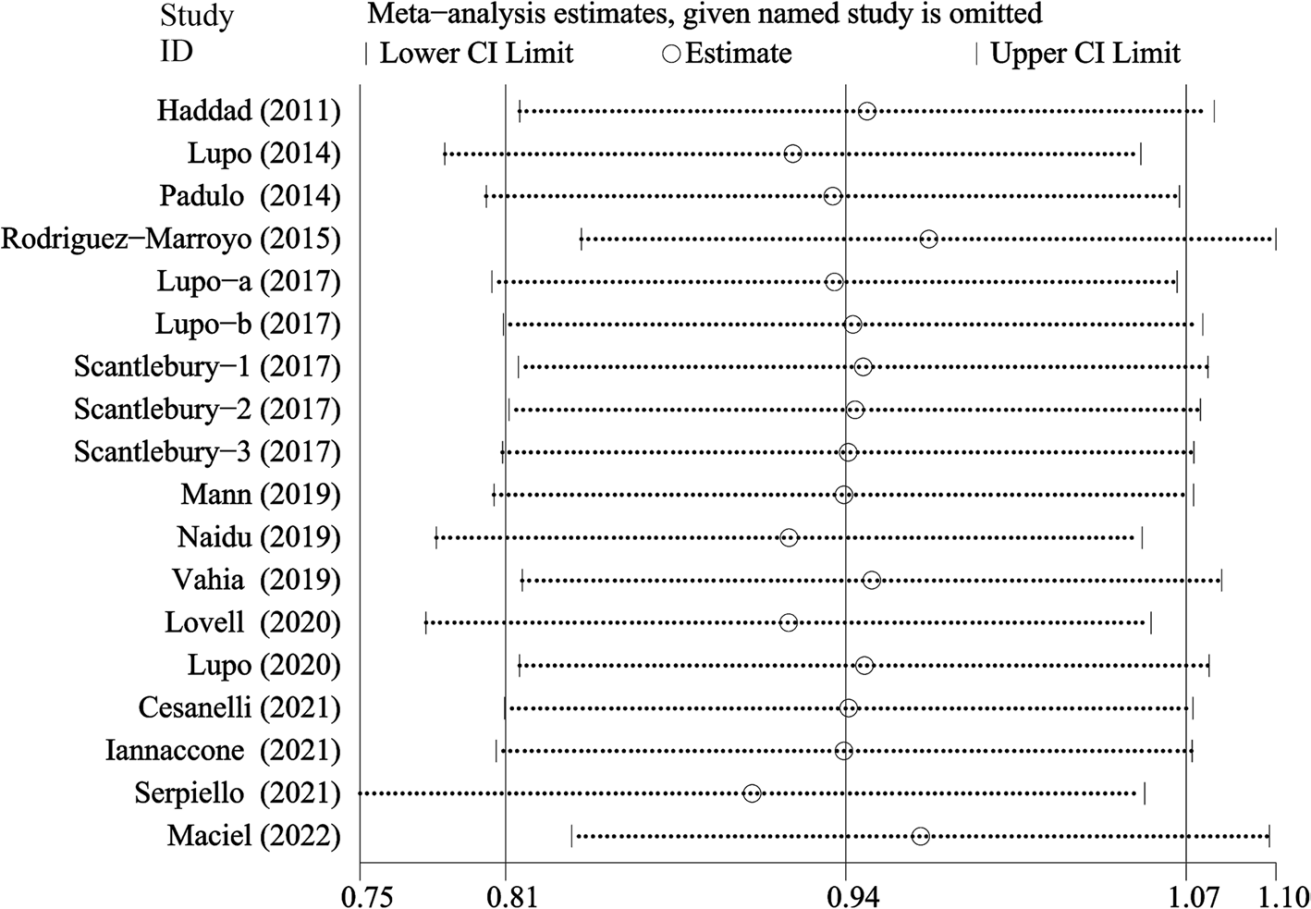
Sensitivity analysis of included studies

The analysis results show that the comprehensive effect size (ES) after excluding the relevant literature one by one is still within the boundary, indicating that the analysis results are stable. A sensitivity analysis was conducted to examine whether there are possible outliers or studies that affect the result (Fig. 3). We found that there are four studies predominantly influencing the result.

**Fig. 4:**
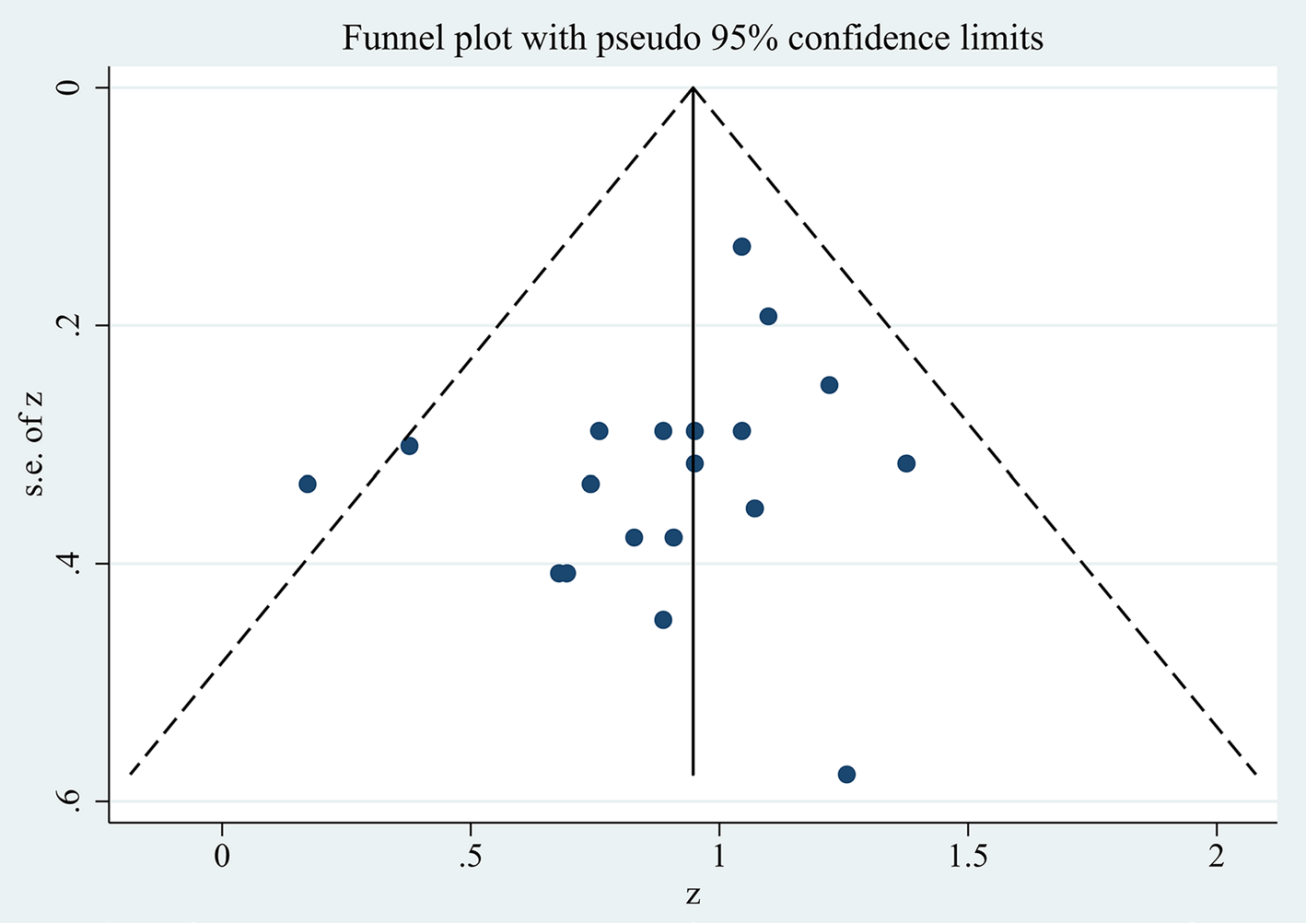
Funnel plot for analyzing the outcome of the rating of perceived exertion scores, which is symmetrical and indicates no bias of included studies

The funnel plot and Egger’s test were used to evaluate the small-study effect and publication bias. Overall, we found no evidence of publication bias in any of the research. According to the funnel plot (Fig. 4), there was no indication of asymmetry or publication bias. The result of Egger’s test suggests that studies had a minimal risk of bias (*p* = 0.161). The Begg’s test also suggests there had no publication bias (*p* = 0.209 > 0.05).

## Discussion

This meta-analysis aimed to characterize the strength of the relationship between internal training load indicator and s-RPE in adolescent athletes. This study first demonstrated moderate to strong overall correlations (*r* = 0.74) between s-RPE and HR measures during exercise. Secondly, it seemed that the s-RPE scale using CR-100 (*r* = 0.80) was more in line with and capable of reflecting the physiological internal load than the CR-10 (*r* = 0.69).

It is noteworthy that the original Borg RPE scale CR-10 was developed for adults while previous studies have shown that adapted RPE scale specific to children and adolescents including intuitive pictorial descriptors could have a better evaluation performance [33]. Although both the CR-10 and CR-100 did not provide clear pictorial descriptors for young people, all studies included in our meta-analysis have provided considerable time of pre-familiarity period in using s-RPE (from 1 week to 1 season) to those junior participants. It is believed to be sufficient in eliminating most scoring bias due to the cognitive differences in most young groups. However, the study from Rodríguez and colleagues, and Maciel et al. have shown an exception in that only very weak to weak (*r* = 0.17–0.36) associations between s-RPE and HR measures were observed [40, 49]. Since the study of Rodríguez et al. focused on children with age under 12, it is speculated that such massive discrepancies between the two metrics were due to the biased or inconsistent scoring from children participants. On the other hand, Maciel et al. did not report any familiarization period. Therefore, the effectiveness of using a 1-month pre-familiarity period for enhancing and optimizing younger children (especially when age < 12) in reflecting their true internal load with CR-10 scale is highly questionable. Likewise, Scantlebury et al. have demonstrated a slightly higher correlation (*r* = 0.72 vs. 0.60) between s-RPE (CR-10 scale) and HR measures in the older group (age = 17.2 vs. 16.7) [48]. Meanwhile, their younger group has shown a much wider range of 95% CI (0.47 to 0.71) and this indicated that some of their younger participants only had a moderate association between two internal TL metrics. Similarly, despite the fairly strong correlations between s-RPE and HR measures when using the CR-100 scale, the study from Serpiello et al. also revealed an increasing trend of correlation (*r* = 0.78–0.84) between s-RPE and HR measures in older subjects (U15 vs. U20) of the young groups [50]. Therefore, further studies to investigate the optimum chronological age in using CR-10 and CR-100 scales as well as the effective measures in enhancing the consistency of s-RPE score from the young groups are warranted.

When comparing the criterion validity between CR-10 and CR-100, after the correlation coefficient transformed to Fisher z-score, our study revealed a relatively stronger association between s-RPE and HR measures in using CR-100 (z = 1.09) than that of CR-10 (z = 0.85). In this regard, a recent study from Fanchini et al. comparing CR-10 and CR-100 using young adult soccer players indicates that CR-100 is a valid measure of internal TL for top-level athletes [51]. Moreover, they suggested that CR-100 and CR-10 scales are interchangeable while the CR-100 can be preferable to the CR-10 scale as it is a more finely graded scale with a wider numerical range and sensitivity but lesser clustering of ratings around the verbal anchors than that of the CR-10. Likewise, Borg and Kaijser also recommended CR-100 as a more accurate scale for similar explanations. Apparently, our study focusing on younger groups also concurred with their findings and suggestions that a better validity in CR-100 over CR-10 was shown. However, due to the very limited number of studies (only 3) and samples (*n* = 108) while only soccer players were included in studies using CR-100, it is premature to conclude if CR-100 is a more preferable scale over CR-10 for children, youth and adolescent athletes in different sports.

The current study has reviewed the findings from most studies using team sports (basketball, soccer, rugby, water polo, handball, beach handball, and field hockey) except the five from Cesanelli et al. (cycling) [46], Mann et al. (Treadmill run) [41], Haddad et al.(Taekwondo athletes) [36], Padulo et al. (Karate) [38], and Lupo et al. (Taekwondo athletes) [22]. The original Borg’s RPE scale was regarded as a valid method to quantify the internal intensity and toughness for a particular exercise regime such as graded running or bike protocols [50]. When the RPE scale was used to quantify the entire training session as s-RPE, it was also shown to be valid in team sports with diversified physical demands (speed, strength, agility, endurance, and neurocognitive) and complex situations (technical and tactical demands) [51]. Interestingly, Chen et al. suggested that the validity between the Borg RPE scale and physiological metrics could be varied due to the sampling groups. They suggested using large heterogeneous samples to enhance both the stability and performance of validity coefficients. In this regard, the low validity observed in the study from Rodríguez-Marroyo et al. (12 male children soccer players) and Maciel et al. (14 male youth handball athletes) can be partially explained by their small homogeneous samples [22, 25]. Conversely, Lupo et al. (only 4 male and 5 female pre-adolescent taekwondo athletes) still showed a strong association between TL monitoring using s-RPE and HR measures [49]. Similarly, a recent systematic review conducted by Rodríguez and colleagues also reported a strong to very strong correlation between RPE and HR in several studies using small samples of children (*n* = 14 to 15). Such discrepancies could be partially explained by the nature of the exercises involved as all participants in the study from Rodríguez et al. only participated in standardized incremental protocols with the bike, running, or stepping modalities in the controlled indoor environment. These continuous aerobic dominant exercises are simple, monotonous, and highly demanding on the cardiorespiratory system while the involvements of other physical qualities such as strength and neurocognitive functions are minimal. Moreover, the temperature and humidity could be fully controlled in lab-based conditions. Since the cardiorespiratory challenge was predominantly reflected by HR, the observed RPE values in these studies could highly reflect and match the internal TL using HR. In this regard, the study of Mann et al. using treadmill runs in our findings concurred with this observation (*r* = 0.74 to 0.89). Likewise, the complexity and dynamics in indoor individual sports, such as taekwondo, are believed to be substantially lesser than in team sports. Therefore, it is speculated that when using s-RPE in investigating the TL of children or adolescent athletes, it can be more forgiving in the sampling (size and groups) processes for indoor individual sports while large heterogeneous samples are required to safeguard good stability and performance of s-RPE in complex team sports such as soccer and rugby.

Although HR was considered a criterion measure of the internal TL while s-RPE using either CR-10 or CR-100 was shown to be valid in TL monitoring in our study, it is worth noting the potential limitations when using these assessment tools. The recent study from Lupo et al. found that s-RPE was more influenced by mostly the total session duration but not the maximal intensity (90% to 100% HR max) and type of training (technique, strength training, or conditioning sessions) for female junior basketball players [45]. It seemed that the s-RPE better reflected the volume component (duration) rather than the intensity part. Since the typical TL calculation using s-RPE was performed by s-RPE × duration, the final TL values could be volume dominant but not equally effective in showing both intensity and duration. Although HR was proved to be a strong indicator to reflect exercise toughness and intensity (e.g. running speed under the aerobic condition), it is noteworthy that the genuine toughness of those female basketball players might not be fully depicted by the use of HR zone (e.g. 90% to 100% predicted HR max as their maximal intensity) in mixed training conditions (conditioning sessions or practices mixed with aerobic and anaerobic components). Therefore, jumping to the conclusion of no emerged effect between s-RPE and the training intensity could also be dogmatic. Despite their inconclusive relationships, it is still speculated that the s-RPE does not fully and truly presenting the training intensity and this could potentially lead to the decrease of criterion validity observed in our studies. Coaches should be cautious when using s-RPE to quantify the training intensity and TL in this regard. On the other hand, the capability of HR in reflecting the TL of anaerobic and strength demands that are inherent to team sports such as soccer is also questionable. Simply relying on HR measures to quantify TL for complex team sports with diversified demands in physical, technical, mental, and tactical components can be misleading. Impellizzeri also demonstrated that HR measures may not adequately reflect the anaerobic and strength demands inherent in team sports like soccer. This is because HR-based methods primarily focus on aerobic effort and may not accurately capture the high-intensity bursts and explosive movements characteristic of these sports [15]. Individuals usually require a few minutes to achieve a steady state of elevated HR to truly reflect the genuine exercise intensity. Therefore, the HR observed during team sports may have a substantial time lag whereas the heart rate values of short explosive moves may also be offset and diluted by prolonged low-intensity tasks interspersed during the game. Considering that the s-RPE method measures both physical and psychological stress, it may be considered a better indicator for measuring global internal TL than HR-based approaches [15].

A few limitations of this study should be highlighted. Firstly, only the correlation coefficients between s-RPE and HR measures were observed and compared while other physiological parameters such as VO_2_, blood lactate, mental stress, and muscle fatigue were not included. The sources and degrees of bias leading to a higher or lower s-RPE value in training sessions were not considered in the current study. Moreover, only a limited number of sports (7 team sports and 4 individual sports) and populations were included, and therefore, our results may not be applicable to other sports and players at different levels. Since only 3 studies about CR-100 were included, it is difficult to conclude if CR-100 is superior to CR-10 for TL monitoring in children and adolescent athletes. For future studies, it is crucial to adopt multivariate approaches that delve into training context, fatigue, and sport-specific performance. When integrating athlete monitoring into a decision-support system, it becomes imperative to address several methodological considerations at each stage of the decision-making process. Accurate measurement and the identification of the smallest meaningful change are essential factors in interpreting individual training responses [52].

## Conclusion

This in-depth systematic review and meta-analysis revealed both CR-10 and CR-100 s-RPE scales as valid tools for TL monitoring in adolescent athletes. Considering the superior benefits of s-RPE and high criterion validity with HR measures, coaches can use it as a "stand-alone" tool for global internal TL monitoring. More future studies on young athletes in different sports using s-RPE on the CR-100 scale are required to con-firm its validity. Meanwhile, further studies for understanding the sources of potential biases in using CR-10 or CR-100 are warranted to enhance the performance of using s-RPE in children and adolescent athletes for TL monitoring. Due to the inconsistent s-RPE pre-familiarity period used in studies, it is difficult to conclude the optimal familiarization duration for young athletes in different age groups.

### Supplementary Information

**Additional file 1: Table S1.** PRISMA checklist.

**Additional file 2: Figure S1.** Certainty assessment (GRADE Pro).

**Additional file 3.**

## Data Availability

The datasets used and/or analyzed during the current study are available from the corresponding author on reasonable request.
