# Supplementary figures and images for: A meta-analysis of the criterion-related validity of Session-RPE scales in adolescent athletes
Source: BMC Sports Sci Med Rehabil. 2023 Aug 12;15:101. doi: 10.1186/s13102-023-00712-5 (PMC10422765; doi:10.1186/s13102-023-00712-5)

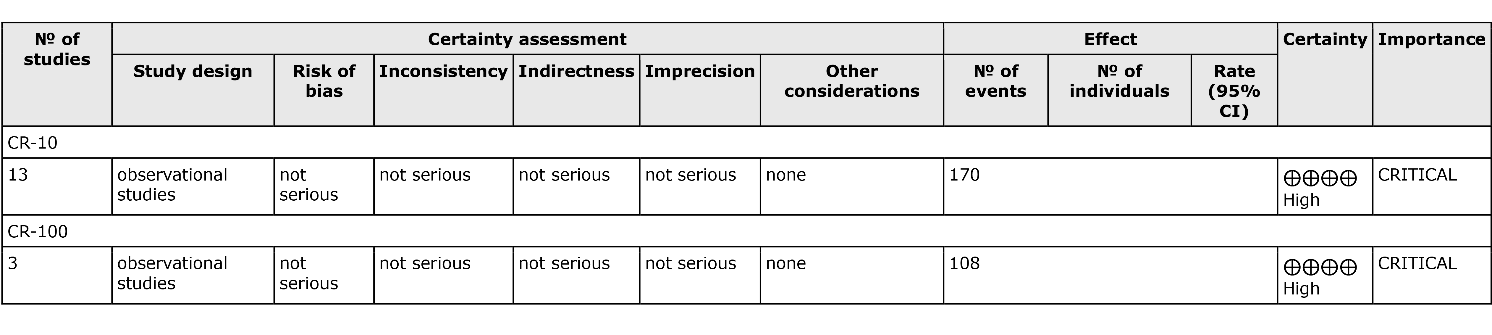
**Figure S1. Certainty assessment (GRADE Pro)**

Supplement: Supplementary file 2 — Additional file 2: Figure S1. Certainty assessment (GRADE Pro). [file 13102_2023_712_MOESM2_ESM.docx]
