# Supplementary material for: A meta-analysis of the criterion-related validity of Session-RPE scales in adolescent athletes
Source: BMC Sports Sci Med Rehabil. 2023 Aug 12;15:101. doi: 10.1186/s13102-023-00712-5 (PMC10422765; doi:10.1186/s13102-023-00712-5)
Supplement: Supplementary file 3 — Additional file 3. [file 13102_2023_712_MOESM3_ESM.docx]

**Search strategy**

Medical Subject Headings (MeSh) (adolescents) and free terms (s-RPE, RPE session, Rating Perceived Exertion session, RPE, Rate of Perceived Exertion, Rated of Perceived Exertion, young, teen*, validity, concurrent validity, correlation) were used. The Boolean operators AND and OR were also used. Different databases search strategies are listed as follow:

1. PubMed

((validity[Title/Abstract]) OR (correlation [Title/Abstract]) OR (concurrent validity[Title/Abstract])) AND ((((((s-RPE[Title/Abstract]) OR (RPE session[Title/Abstract])) OR (Rating Perceived Exertion session[Title/Abstract])) OR (RPE[Title/Abstract])) OR (Rate of Perceived Exertion[Title/Abstract])) OR (Rated of Perceived Exertion[Title/Abstract]) AND (((("Adolescent"[Mesh]) OR (young[Title/Abstract])) OR (teen*[Title/Abstract])) OR (youth)))

1. WOS

(TS=(RPE session OR Rating Perceived Exertion session OR s-RPE OR Rate of Perceived Exertion OR Rated of Perceived Exertion OR RPE)) AND (TS=(Adolescent OR Teen* OR Youth OR young)) AND (TS=(validity OR concurrent validity OR correlation))

1. Cochrane

(RPE session):ab,ti,kw OR (Rating Perceived Exertion session):ab,ti,kw OR (s-RPE):ab,ti,kw OR (Rate of Perceived Exertion):ab,ti,kw OR (Rated of Perceived Exertion):ab,ti,kw OR (RPE) :ab,ti,kw

(Adolescent):ab,ti,kw OR (Teen*):ab,ti,kw OR (Youth):ab,ti,kw OR (young):ab,ti,kw

(validity):ab,ti,kw OR (concurrent validity):ab,ti,kw

Mesh: adolescent

1. Embase

'adolescent'/exp

'rpe session':ab,ti OR 'rating perceived exertion session':ab,ti OR 's-rpe':ab,ti OR 'Rate of Perceived Exertion':ab,ti OR 'Rated of Perceived Exertion':ab,ti OR 'RPE':ab,ti

'adolescent':ab,ti OR 'teen*':ab,ti OR 'youth':ab,ti OR 'young':ab,ti


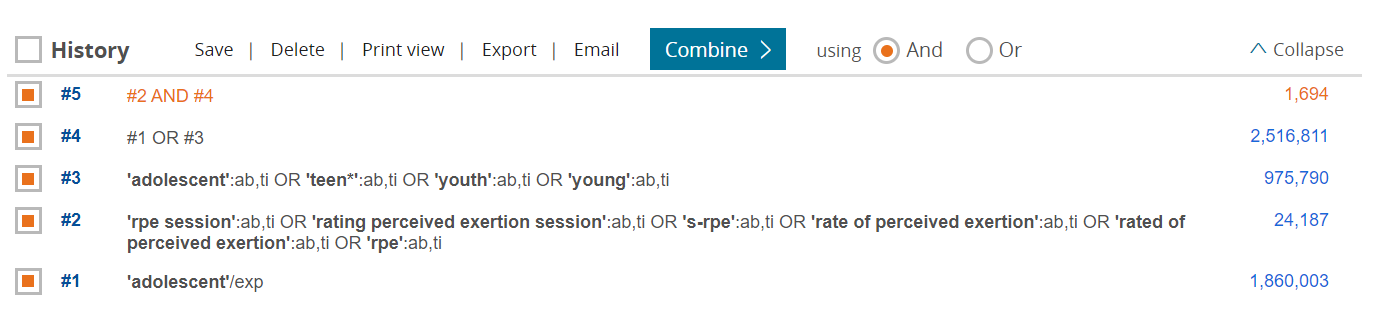


'validity':ab,ti OR 'concurrent validity':ab,ti OR 'correlation':ab,ti

1. EBSCO

#1 RPE session OR Rating Perceived Exertion session OR s-RPE OR RPE OR Rate of Perceived Exertion OR Rated of Perceived Exertion

#2 Adolescent OR Teen* OR Youth OR Young

#1 AND #2

#3 validity OR concurrent validity OR correlation

1. OVID

| Set | Search Statement |
| --- | --- |
| 1. | (RPE session or Rating Perceived Exertion session or s-RPE or RPE or Rate of Perceived Exertion or Rated of Perceived Exertion).ti,ab,kw. |
| 2. | (Adolescent or Teen* or Youth or Young).ti,ab,kw. |
|  | (validity or concurrent validity or correlation).ti,ab,kw. |
| 3. | 1 and 2 |

1. Scopus：

TITLE-ABS-KEY ( "RPE session" OR "Rating Perceived Exertion session" OR "s-RPE" OR "RPE" OR "Rated of Perceived Exertion" OR "Rate of Perceived Exertion" ) AND TITLE-ABS-KEY ( "Adolescent" OR "Teen*" OR "Youth" OR "Young" ) AND TITLE-ABS-KEY ( "validity" OR "concurrent validity" OR "correlation")

1. Proquest

("validity" OR "correlation" OR "concurrent validity") AND ("s-RPE" OR "RPE session" OR "Rating Perceived Exertion session" OR "RPE" OR "Rate of Perceived Exertion" OR "Rated of Perceived Exertion") AND ("Adolescent" OR "young" OR "teen*" OR "youth")
